# Pan-Phosphodiesterase Inhibitors Attenuate TGF-β-Induced Pro-Fibrotic Phenotype in Alveolar Epithelial Type II Cells by Downregulating Smad-2 Phosphorylation

**DOI:** 10.3390/ph15040423

**Published:** 2022-03-30

**Authors:** Katarzyna Wójcik-Pszczoła, Grażyna Chłoń-Rzepa, Agnieszka Jankowska, Bruno Ferreira, Paulina Koczurkiewicz-Adamczyk, Elżbieta Pękala, Elżbieta Wyska, Krzysztof Pociecha, Reinoud Gosens

**Affiliations:** 1 Department of Pharmaceutical Biochemistry, Faculty of Pharmacy, Jagiellonian University Medical College, Medyczna 9, 30-688 Kraków, Poland; bruno.fer.9719@gmail.com (B.F.); paulina.koczurkiewicz@uj.edu.pl (P.K.-A.); elzbieta.pekala@uj.edu.pl (E.P.); 2 Department of Medicinal Chemistry, Faculty of Pharmacy, Jagiellonian University Medical College, Medyczna 9, 30-688 Kraków, Poland; mfchlon@cyf-kr.edu.pl (G.C.-R.); agnieszkajankowska@poczta.onet.pl (A.J.); 3 Department of Pharmacokinetics and Physical Pharmacy, Faculty of Pharmacy, Jagiellonian University Medical College, Medyczna 9, 30-688 Kraków, Poland; mfwyska@cyf-kr.edu.pl (E.W.); k.pociecha@uj.edu.pl (K.P.); 4 Department of Molecular Pharmacology, University of Groningen, Antonius Deusinglaan 1, 9713 AV Groningen, The Netherlands; r.gosens@rug.nl

**Keywords:** phosphodiesterase inhibitors, alveolar epithelial type II cells, transforming growth factor type β, asthma, lung fibrosis, airway remodeling, mesenchymal-like phenotype, cell migration, TGF-β signaling

## Abstract

Airway remodeling is a pathological process that accompanies many chronic lung diseases. One of the important players in this process are epithelial cells, which under the influence of pro-inflammatory and pro-fibrotic factors present in the airway niche, actively participate in the remodeling process by increasing extracellular matrix secretion, acquiring migration properties, and overproducing pro-fibrotic transducers. Here, we investigated the effect of three new 8-arylalkylamino- and 8-alkoxy-1,3-dimethyl-2,6-dioxo-1,2,3,6-tetrahydro-7*H*-purin-7-yl-*N*-(5-(*tert*-butyl)-2-hydroxyphenyl)butanamides (**1**, **2**, and **3**), representing prominent pan-phosphodiesterase (pan-PDE) inhibitors on transforming growth factor type β (TGF-β)-induced alveolar epithelial type II cells (A549 cell line) of a pro-fibrotic phenotype. Our results demonstrate for the first time the strong activity of pan-PDE inhibitors in the prevention of TGF-β-induced mesenchymal markers’ expression and A549 cells’ migration. We also showed an increased p-CREB and decreased p-Smad-2 phosphorylation in TGF-β-induced A549 cells treated with **1**, **2**, and **3** derivatives, thereby confirming a pan-PDE inhibitor mesenchymal phenotype reducing effect in alveolar epithelial type II cells via suppression of the canonical Smad signaling pathway. Our observations confirmed that PDE inhibitors, and especially those active against various isoforms involved in the airway remodeling, constitute an interesting group of compounds modulating the pro-fibrotic response of epithelial cells.

## 1. Introduction

It is widely accepted that epithelial cells are actively involved in repairing injured tissue [1]. Aberrantly stimulated, they may secrete a large amount of pro-inflammatory and pro-fibrotic factors, as well as extracellular matrix (ECM) components, whose excessive production and deposition is connected with tissue fibrosis. Moreover, the overproduction of interleukins, chemokines, and growth factors by epithelial cells directly affects their mesenchymal counterparts, including fibroblasts and myofibroblasts, both known to comprehensively orchestrate the process of tissue fibrosis [2,3,4,5,6,7]. On the other hand, a vast variety of pro-inflammatory and pro-fibrotic stimuli make epithelial cells able to migrate, secrete ECM, and move to a more mesenchymal phenotype. Disruptions in epithelial cells’ functions may contribute to liver, kidney, heart, intestinal, ocular, and adipose tissue fibrosis, as well as scleroderma [1,8]. An increasing number of studies have demonstrated that epithelial cells developing mesenchymal characteristics play an important role in airway remodeling in asthma as well as contribute to lung fibrosis in chronic obstructive lung disease (COPD) or idiopathic pulmonary fibrosis (IPF) progression [1,8,9,10,11]. Since it has been emphasized that airway epithelial cells can actively drive the process of tissue fibrosis, they are now intensively investigated as novel therapeutic approaches to limit lung fibrosis. So far, several different factors that may contribute towards mesenchymal properties of epithelial cells have been identified, including pleiotropic growth factors and, among them, one of the best known pro-fibrotic ones, transforming growth factor type β (TGF-β). TGF-β has been shown to induce the pro-fibrotic phenotype in various types of respiratory epithelial cells, including human bronchial epithelial cells and alveolar epithelial cells [9,12,13].

Lately, a large body of evidence points to phosphodiesterase (PDE) inhibitors as promising anti-inflammatory and anti-fibrotic agents [14,15,16,17,18]. Phosphodiesterases are cellular enzymes responsible for the breakdown of the secondary signaling transducers—adenosine 3′,5′-cyclic monophosphate (cAMP) and guanosine 3′,5′-cyclic monophosphate (cGMP). Therefore, their inhibitors lead to an increase in the intracellular cAMP and/or cGMP level. Numerous studies have shown the anti-fibrotic actions of cAMP, including a decrease in fibroblasts’ proliferation, migration, and transition into myofibroblasts, as well as inhibition of pro-fibrotic growth factors and ECM production [19]. It is also known that both PDE inhibitors and elevated intracellular cAMP may diminish the mesenchymal phenotype in diverse pulmonary cells of epithelial origin [14,19]. This is of particular interest given the increased expression of some PDE isoforms, e.g., PDE4 and PDE8, accompanying the acquisition of the mesenchymal phenotype by alveolar type II cells [20].

**Figure 1:**
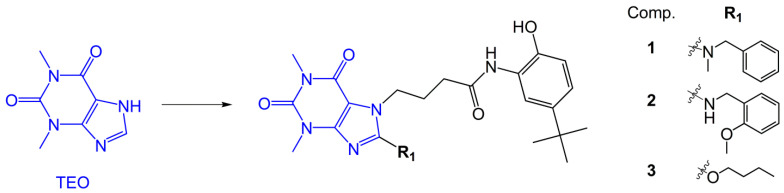
Chemical structures of study compounds: 1,3-dimethyl-3,7-dihydro-1*H*-purine-2,6-dione (TEO) and 8-arylalkylamino (**1**, **2**) and 8-alkoxy (**3**) derivatives of 1,3-dimethyl-2,6-dioxo-1,2,3,6-tetrahydro-7*H*-purin-7-yl-*N*-(5-(*tert*-butyl)-2-hydroxyphenyl)butanamide.

**Figure 2:**
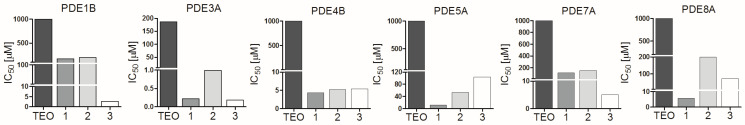
Graphical representation of IC_50_ values (μM) of **1**, **2**, **3**, and TEO against selected PDEs. The IC_50_ values for PDE1B, 3A, 4B, 5A, 7A, and 8A were published in [23,25], except PDE8A IC_50_ for TEO, which was determined in this study and was over 1000 μM.

Recently, we have synthesized a large group of 7,8-disubstituted 1,3-dimethyl-3,7-dihydro-1*H*-purine-2,6-dione (theophylline, TEO) derivatives [21,22,23]. These compounds, in contrast to TEO, which is a prototype and non-selective PDE inhibitor, were able to inhibit individual PDE isoforms at the nano and/or micromolar level [23,24,25]. Their high inhibitory activity against selected PDEs was translated into promising anti-inflammatory and anti-fibrotic activity in vitro. Selected 7,8-disubstituted 1,3-dimethyl-3,7-dihydro-1*H*-purine-2,6-dione derivatives significantly reduced the TGF-β-induced pro-fibrotic phenotype of human lung fibroblasts and airway smooth muscle cells [24,25]. Our pan-PDE inhibitors were also able to diminish several inflammatory and remodeling responses in interleukin 13 (IL-13) or TGF-β-induced human bronchial epithelial cells isolated from mild asthmatics [23]. However, their effect on TGF-β-induced acquisition of the mesenchymal phenotype by epithelial cells remained unknown. Thus, the current study was designed to explore the potential pan-PDE inhibitors’ activity against TGF-β-induced human alveolar epithelial type II cells (A549 cell line). To verify this hypothesis, we selected three, representative and most active in previous research, compounds: 8-arylalkylamino (**1**, **2**) and 8-alkoxy (**3**) derivatives of 1,3-dimethyl-2,6-dioxo-1,2,3,6-tetrahydro-7*H*-purin-7-yl-*N*-(5-(*tert*-butyl)-2-hydroxyphenyl)butanamide (Figure 1), representing differential inhibitory activity against individual PDE isoforms (Figure 2), and compared their effect with TEO, a well-known, non-selective PDE inhibitor. To achieve this goal, we analyzed the expression of some mesenchymal markers (e.g., cadherin, vimentin, collagen I, fibronectin, snail transcription factor), as well as the phenotype and migration of TGF-β-induced alveolar epithelial type II cells cultured in the presence of the tested compounds.

## 2. Results

### 2.1. Pan-PDE Inhibitors Slightly Modulate Basic A549 Cells’ Functions

**Figure 3:**
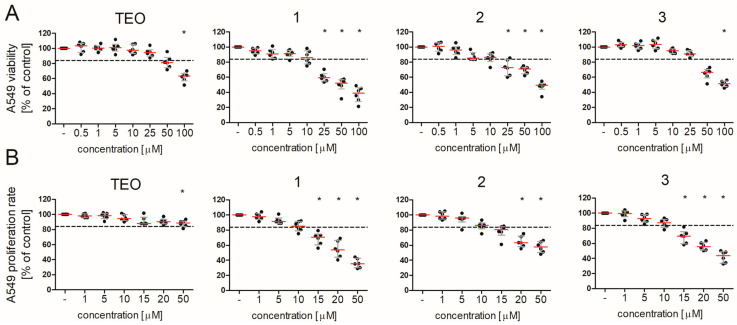
Pan-PDE inhibitors affect A549 cells’ viability and proliferation rate. A549 cells were cultured overnight and then incubated in growing concentrations of **1**, **2**, **3**, and TEO. Cell viability was determined by the MTT assay (**A**) and the cell proliferation rate was assessed by crystal violet nuclei staining (**B**) after 48 h of incubation with the tested compounds. Horizontal line represents the median with interquartile range (in comparison to non-treated cells normalized to 100%), *n* = 6 (Kruskal–Wallis test with Dunn’s post-hoc test). The dashed lines mark the values above which the tested compounds were considered non-toxic (**A**) and did not affect cell proliferation (**B**). The results were considered statistically significant at the *p*-level of 0.05 (*).

8-Substituted 1,3-dimethyl-2,6-dioxo-1,2,3,6-tetrahydro-7*H*-purin-7-yl-*N*-(5-(*tert*-butyl)-2-hydroxyphenyl)butanamides (**1**, **2**, and **3**) have not been investigated in the alveolar epithelial type II cell line A549 so far. Therefore, the aim of the preliminary studies was to analyze the effects of these pan-PDE inhibitors on cell functions, including viability and proliferation rate. A549 cell viability was reduced in the presence of **1**, **2**, and **3** in a concentration-dependent manner (Figure 3A). A significant decrease in viability was noted for cells cultured in the medium containing **1** and **2** at concentrations above 25 µM (by 42% and 28%, respectively), and **3** and TEO at 100 µM (by 48% and 38%, respectively) (Figure 3A). Thus, 8-alkoxy derivative (**3**) and TEO showed a better cellular toxicity profile in A549 cells than both 8-arylalkylamino derivatives (**1** and **2**).

A similar association was not observed for the proliferation analysis. In this case, compounds **1**–**3** at concentrations above 10 µM similarly inhibited the A549 proliferation rate (Figure 3B). A slight cytostatic effect was demonstrated for TEO at the highest applied concentration (Figure 3B). Based on these preliminary results, further analyses were performed with all tested pan-PDE inhibitors **1**–**3** administrated at a concentration of 10 µM, as this concentration was found to be non-toxic (viability was at least 85%, Figure 3A dashed line) and did not significantly reduce the cell proliferation rate (by more than 16%, Figure 3B dashed line).

### 2.2. TGF-β-Induced Mesenchymal-like Phenotype in A549 Cells Is Diminished by Pan-PDE Inhibitors

**Figure 4:**
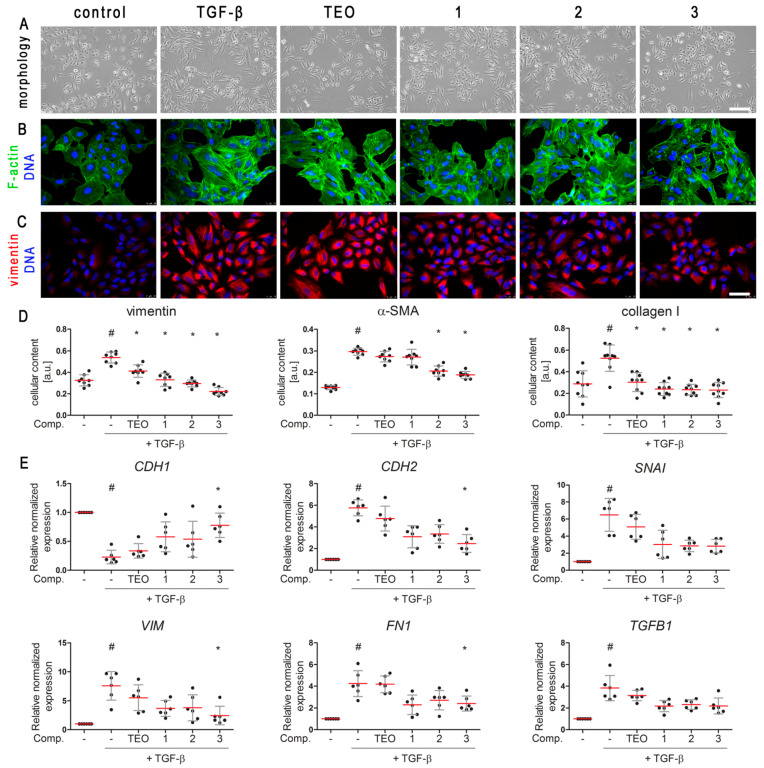
Pan-PDE inhibitors decrease the TGF-β-induced pro-fibrotic phenotype in alveolar epithelial type II cells. A549 cells were cultured overnight and then serum-deprived for 24 h. **1**, **2**, **3**, or TEO (10 μM) was administrated 1 h before TGF-β (5 ng/mL) treatment for 24 h (**E**) or 48 h (**A**–**D**) of incubation. (**A**) Representative images (100× magnification) illustrating TGF-β-induced A549 morphology in the presence of the tested compounds. Scale bar = 200 µm. (**C**,**B**) Representative images (400× magnification) of A549 cells stained for F-actin and nuclei (**B**) and immunostained for vimentin and nuclei (**C**). Cells were fixed, permeabilized, blocked, and incubated with anti-vimentin antibody followed by an Alexa Fluor 546 conjugated antibody or TRITC-labeled phalloidin, and nuclei were co-stained with Hoechst 33342 dye. Scale bar = 50 µm. (**D**) Cellular content of vimentin, α-SMA, and collagen type I was determined by in-cell ELISA. Horizontal lines represent the mean value (±SD), *n* = 8 (ANOVA with Dunnett’s post-hoc test). (**E**) Relative, normalized to *18S rRNA* level, expression of *CDH1*, *CDH2*, *SNAI*, *VIM*, *FN1*, and *TGFB1* was carried out by RT-qPCR analysis. Horizontal lines represent the median with interquartile range, *n* = 6 (Kruskal–Wallis test with Dunn’s post-hoc test). The results were considered statistically significant at the *p*-level of 0.05, compared to basal control (#) and TGF-β (*) conditions.

In addition to the well-established involvement of TGF-β in triggering epithelial cells to acquire mesenchymal features, this growth factor is a major player in the pathogenesis of chronic lung diseases [26,27]. Therefore, the next step was to understand the influence of 8-arylalkylamino (**1** and **2**) and 8-alkoxy (**3**) derivatives, representing pan-PDE inhibitors, on the TGF-β-induced mesenchymal features in A549 cells. Alveolar epithelial type II cells’ phenotype showed a flattened and spindle-shaped morphology after culture in the presence of TGF-β (Figure 4A). This characteristic mesenchymal-like shape of the cells changed towards more epithelial-like after the cells were preincubated in the presence of **1**, **2**, and **3** (Figure 4A). The observed change in cell morphology was also reflected in the cell elongation index (EI, the ratio between the major and minor axis of the cell) measurement. The calculated EI was 0.578 in TGF-β-induced cells (significant growth in comparison to control cells’ EI—0.138). A meaningful reduction of the EI value to 0.182, 0.241, 0.178, and 0.372 in TGF-β-induced cells cultured in the presence of **1**, **2**, **3**, and TEO, respectively, was demonstrated. The actin cytoskeleton labeling showed that **1**, **2**, and **3**, in TGF-β-induced cells, partially restored the original, epithelial-like organization, present in control cells (Figure 4B). A reduction in the number of visible and elongated stress fibers was observed in the TGF-β-induced cell populations treated with the evaluated compounds **1**–**3** (Figure 4B). In the case of TGF-β-induced cells cultured in the presence of TEO, redistribution of actin stress fibers was not as strongly pronounced as with the tested pan-PDE inhibitors.

The observed activity of the tested compounds prompted us to undertake further analysis of markers, the presence of which may indicate that epithelial cells acquire mesenchymal features [1,28]. In-cell ELISA revealed a strong induction of vimentin, α-smooth muscle actin (α-SMA), and collagen I in TGF-β-induced A549 cells (Figure 4D). As demonstrated in Figure 4D, pan-PDE inhibitors **1**–**3** diversified diminished TGF-β-induced mesenchymal proteins in alveolar epithelial type II cells. Their inhibitory effect was particularly evident in relation to vimentin and collagen I (Figure 4D). As shown in the micrographs (Figure 4C), the reducing effect of **1**, **2**, and **3** was also reflected in the amount of vimentin in individual A549 cells, while for TEO, no significant inhibition of TGF-β-induced vimentin was observed. As the change in cadherin expression contributes to the development of mesenchymal characteristics in epithelial cells, we also examined the E-cadherin and *N*-cadherin gene expression level in TGF-β-treated A549 cells. While *CDH1* expression was significantly lowered after TGF-β, *CDH2* expression was increased almost 6-fold (Figure 4E). The loss of the E-cadherin transcript level in TGF-β-treated cells was restored by the pan-PDE inhibitors, and the effect was particularly evident for **1** and **3** derivatives (Figure 4E). Conversely, the TGF-β-elevated *N*-cadherin transcript level was reduced at least twice in the presence of all three tested derivatives (Figure 4E). Subsequently, an implicated E-cadherin repression transcription factor, *SNAI*, was highly elevated after TGF-β, whereas in A549 populations cultured in the presence of pan-PDE inhibitors **1**–**3**, its expression was markedly diminished. While a 6-fold increase in *SNAI* expression was shown after TGF-β treatment, **1**, **2**, and **3** caused a 2.15-, 2.28-, and 2.3-fold reduction of *SNAI* expression in TGF-β-induced A549 cells, respectively (Figure 4E). Similar properties of **1**, **2**, and **3** were also noted for other mesenchymal-related genes, including *VIM*, *FN1*, and *TGFB1* (Figure 4E). Of all the compounds, TEO has a weak or negligible ability to reduce alveolar epithelial type II cells’ changes towards a mesenchymal phenotype (Figure 4).

### 2.3. TGF-β-Induced A549 Cells’ Migration Is Decreased in Response to Pan-PDE Inhibitors

**Figure 5:**
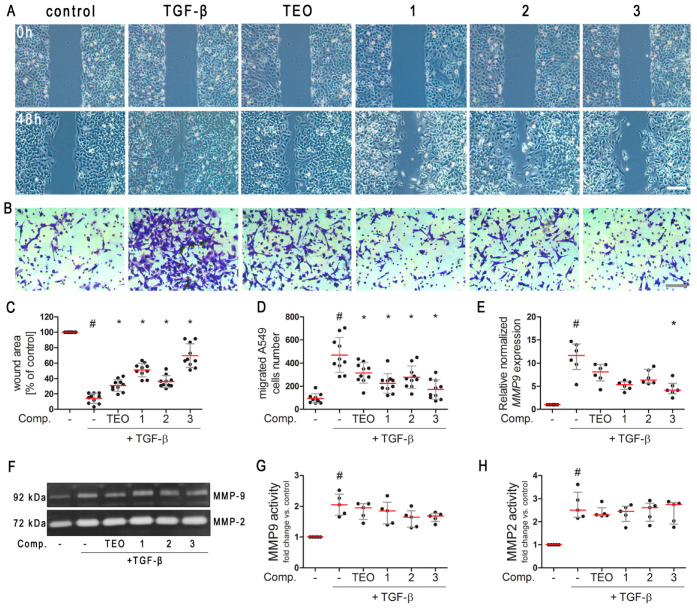
Pan-PDE inhibitors diminish TGF-β-induced migration of alveolar epithelial type II cells. A549 cells were cultured overnight and then serum-deprived for 24 h. **1**, **2**, **3**, or TEO (10 μM) was administrated 1 h before TGF-β (5 ng/mL) treatment for 48 h incubation. Cell migration was examined via the wound healing assay (**A**,**C**) and the Transwell system (**B**,**D**). (**A**) Representative microphotographs (100× magnification) illustrating the wound area at the beginning of the experiment (0 h) and after 48 h of TGF-β-induced A549 cells’ incubation in the presence of **1**, **2**, **3**, or TEO. Scale bar = 200 µm. (**C**) Quantification of wound area in comparison to non-treated control cells after 48 h incubation. Horizontal lines represent the mean value (±SD), *n* = 10 (ANOVA with Dunnett’s post-hoc test). (**B**) Representative microphotographs (100× magnification) demonstrating migrated A549 cell numbers stained with crystal violet. Scale bar = 200 µm. (**D**) Migrated cells were counted in randomly selected fields of view. Horizontal lines represent the mean value (±SD), *n* = 10 (ANOVA with Dunnett’s post-hoc test). (**E**) Expression of *MMP9* gene in comparison to *18sRNA* was quantified by RT-qPCR analysis. Horizontal lines represent the median with interquartile range, *n* = 6 (Kruskal–Wallis test with Dunn’s post-hoc test). (**F**) Representative zymogram showing MMP2 and MMP9 enzymatic activity. (**G**,**H**) Quantification of MMP9 (**G**) and MMP2 (**H**) corresponding bands, optical density normalized to basal control conditions. Horizontal lines represent the median with interquartile range, *n* = 5 (Kruskal–Wallis test with Dunn’s post-hoc test). The results were considered statistically significant at the *p*-level of 0.05, against the control (#) and TGF-β (*) conditions.

Loss of cell–cell adhesion, related mostly to cellular E-cadherin suppression, is one of the causes of excessive epithelial cell migration [29]. Knowing that the tested 1,3-dimethyl-2,6-dioxo-1,2,3,6-tetrahydro-7*H*-purin-7-yl-*N*-(5-(*tert*-butyl)-2-hydroxyphenyl)butanamides **1**, **2**, and **3** significantly reduce the A549 mesenchymal-like phenotype, in the next step we determined their effect on TGF-β-induced alveolar epithelial type II cell migration. To achieve this goal, we used two different models: a wound healing assay and a Transwell system. As expected, TGF-β significantly enhanced 2D, collective A549 migration (Figure 5A). The 48 h A549 incubation with TGF-β resulted in a 7-fold reduction of the wound area when compared to the control (Figure 5C). In turn, all tested compounds, including TEO, significantly increased the observed wound area in TGF-β-treated cells (Figure 5A,C). A similar effect was also demonstrated in the second experimental model. The ability of A549 cells to move through Transwell pores was significantly increased in the presence of TGF-β and was reduced after exposure to 8-substituted derivatives **1**, **2**, and **3** as well as TEO (Figure 5B,D). Both analyses revealed that the tested pan-PDE inhibitors significantly reduced TGF-β-induced alveolar epithelial type II cell migration, but the strongest effect was shown for **1** and **3** (Figure 5A–D).

In order to investigate the potential involvement of matrix metalloproteinase 9 (MMP9) in the pan-PDE inhibitors’ effect on TGF-β-induced A549 migration, we next examined the *MMP9* expression level as well as the activity of this metalloproteinase. While both an increase in the *MMP9* transcript level and MMP9 activity were observed in TGF-β-induced cells, the tested derivatives **1**, **2**, and **3** decreased *MMP9* expression but not its activity (Figure 5E–H, Supplementary Figure S1). As demonstrated in Figure 5G, a slight decrease in MMP9 activity could be observed in TGF-β-induced A549 cells treated with **2** and **3**. In turn, the TGF-β-induced MMP2 activity remained unchanged in the presence of the tested derivatives **1**, **2**, and **3** (Figure 5H).

### 2.4. Pan-PDE Inhibitors Activate CREB Phosphorylation in TGF-β-Induced A549 Cells

**Figure 6:**
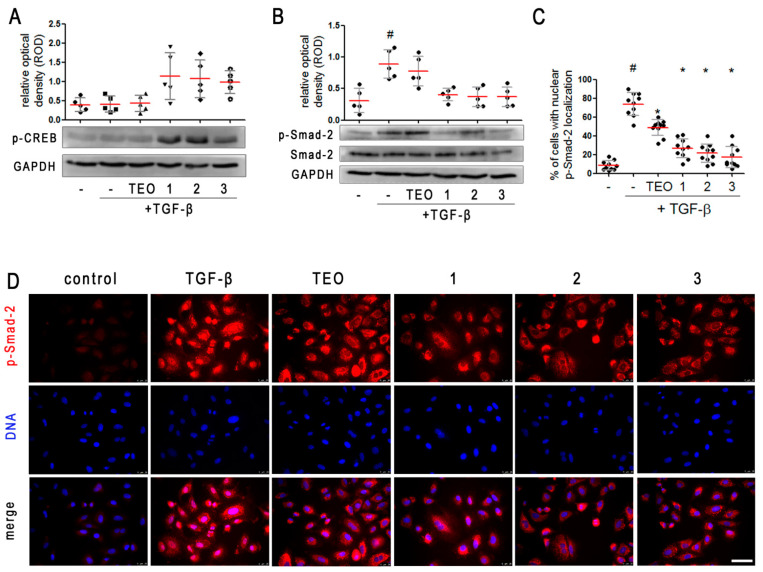
Pan-PDE inhibitors attenuate TGF-β-induced Smad-2 signaling in alveolar epithelial type II cells. A549 cells were cultured overnight and then serum-deprived for 24 h. **1**, **2**, **3**, or TEO (10 μM) was administrated 1 h before TGF-β (5 ng/mL) treatment for an additional 1 h incubation. Representative immunoblots and densitometry analysis of p-CREB (**A**) and p-Smad-2 (**B**) in A549 (*n* = 5). The relative optical density was normalized to the GAPDH control level. Horizontal lines represent the median with interquartile range (Kruskal–Wallis test with Dunn’s post-hoc test). (**D**) Representative images (400× magnification) of A549 immunostained for p-Smad-2. Cells were fixed, permeabilized, blocked, and incubated with anti-p-Smad-2 antibody followed by an Alexa Fluor 546 conjugated antibody, and nuclei were co-stained with Hoechst 33342 dye. Scale bar = 50 µm. (**C**) Cells exhibiting colocalization of p-Samd-2 and nuclear signal were counted in randomly selected fields of view and expressed as a percentage fraction in the entire A549 population. Horizontal lines represent the mean value (±SD), *n* = 10 (ANOVA with Dunnett’s post-hoc test). The results were considered statistically significant at the *p*-level of 0.05, against the control (#) and TGF-β (*) conditions.

One of the most important pathways activated in response to elevated intracellular cAMP, also with reference to PDE inhibitors, is cAMP-response element binding protein (CREB) phosphorylation and cAMP-dependent gene expression. Thus, the next step was to evaluate whether, under the influence of the tested 8-substituted 1,3-dimethyl- 2,6-dioxo-1,2,3,6-tetrahydro-7*H*-purin-7-yl-*N*-(5-(*tert*-butyl)-2-hydroxyphenyl)butanamides, CREB undergoes phosphorylation in TGF-β-induced A549 cells. We found that alveolar epithelial type II cells’ induction with TGF-β alone did not affect CREB phosphorylation (Figure 6A, Supplementary Figure S2). In turn, treatment of TGF-β-induced cells with **1**, **2**, and **3** caused a large increase in CREB phosphorylation on Ser-133 (Figure 6A, Supplementary Figure S2), confirming cAMP-dependent signaling activation in pan-PDE inhibitors-treated A549 cells. This kind of effect was not demonstrated in TGF-β-induced A549 populations cultured in the presence of TEO, a non-selective PDE inhibitor, possibly due to its poor inhibitory activity against individual PDE isoforms.

### 2.5. Pan-PDE Inhibitors Attenuate Smad-2 Signaling in TGF-β-Induced Alveolar Epithelial Type II Cells

Knowing that the key signaling pathway activated in response to TGF-β involves Smad proteins, we next analyzed the contribution of Smad-2 to the attenuation of the mesenchymal-like phenotype in alveolar epithelial type II cells exerted by the tested **1**–**3** pan-PDE inhibitors. Firstly, we determined the Smad-2 phosphorylation level in TGF-β-induced A549 cells preincubated with three tested 8-substituted derivatives. Immunoblotting revealed a decrease of Smad-2 phosphorylation in TGF-β-induced alveolar epithelial type II cells cultured in the presence of **1**, **2**, and **3** (Figure 6B, Supplementary Figure S2). No differences were observed in the activity of individual pan-PDE inhibitors, but TEO had the weakest effect on Smad-2 phosphorylation (Figure 6B, Supplementary Figure S2). Then, we decided to check whether the assessed level of p-Smad-2 correlates with its translocation to the nucleus. Immunostaining of A549 cells cultured in the presence of TGF-β showed a significant increase in both the amount of p-Smad-2 as well as a higher percentage of cells representing the nuclear localization of this protein (79%) (Figure 6C,D). The percentage of cells in which translocation of p-Smad-2 to the nucleus occurred was significantly lower in TGF-β-induced A549 cells cultured in the presence of the tested 8-substituted 1,3-dimethyl-2,6-dioxo-1,2,3,6-tetrahydro-7*H*-purin-7-yl-*N*-(5-(*tert*-butyl)-2-hydroxyphenyl)butanamides (Figure 6C,D). The largest decrease was noted for TGF-β-induced alveolar epithelial type II cells treated with **1**, **2**, and **3**, where the percentage of cells demonstrating p-Smad-2 nuclear localization was 25%, 22%, and 18%, respectively (Figure 6C).

## 3. Discussion

Airway remodeling and lung fibrosis are major features in the pathogenesis of asthma, chronic obstructive lung disease, and pulmonary fibrosis [30,31]. Both arise as a result of multicellular processes occurring in respiratory cells and various immune cells infiltrating bronchi and/or lungs during the course of the disease. Moreover, diversified and pleiotropic cytokines, chemokines, and growth factors secreted by these cells are also involved in airway remodeling [32,33]. Due to the multifaceted and complex nature, lung fibrosis constitutes a serious clinical problem and a still unresolved therapeutic challenge. In the recent years, many attempts have been made to search for anti-fibrotic drugs, both in the group of already known and used substances, as well as in entirely new groups of chemical compounds [34,35,36,37]. Lately, there has been a growing interest in selective or dual PDE inhibitors, especially active against the PDE3, PDE4, PDE5, or PDE7 and PDE8 isoforms, as therapeutic targets for respiratory diseases [14,18]. Some of them, e.g., roflumilast, sildenafil, or tadalafil, have already been used in the clinic, and others, which are mainly inhaled PDE4 and/or PDE3 inhibitors, are intensively evaluated in numerous clinical trials [16].

Our previous research revealed that 7,8-disubstituted 1,3-dimethyl-3,7-dihydro-1*H*- purine-2,6-diones, representing potent pan-PDE inhibitors, are remarkably effective in reducing the fibrotic phenotype of numerous respiratory cells, including human airway smooth muscle cells [24], lung fibroblasts [25], and bronchial epithelial cells derived from asthmatics [23]. The current study aimed to identify the influence of three representative pan-PDE inhibitors on the TGF-β-induced, pro-fibrotic phenotype of alveolar epithelial type II cells, that may contribute to the development of airway remodeling and lung fibrosis. The compounds selected for this study were previously described as very strong inhibitors of individual PDE isoforms (Figure 2), with IC_50_ values comparable to those of selective PDE inhibitors [23,24,25]. The performed analyses allowed us to demonstrate for the first time that various 8-substituted 1,3-dimethyl-2,6-dioxo-1,2,3,6-tetrahydro-7*H*-purin-7-yl-*N*-(5-(*tert*-butyl)-2-hydroxyphenyl)butanamides constitute distinguished compounds limiting several A549 cells’ features associated with their pro-fibrotic phenotype. These potent pan-PDE inhibitors were able to inhibit TGF-β-induced expression of several markers, including vimentin, fibronectin, collagen I, α-smooth muscle actin, *N*-cadherin, and snail-1 transcription factor in alveolar epithelial type II cells. We have also demonstrated that TGF-β-induced migration of these cells is significantly reduced by 8-arylalkylamino and 8-alkoxy derivatives. Moreover, we found that the activity of these pan-PDE inhibitors against TGF-β-treated alveolar epithelial type II cells is a result of their influence on canonical signaling via Smad proteins.

Several different studies have displayed that the selective PDE4 inhibitor—roflumilast *N*-oxide—may reduce epithelial–mesenchymal transition (EMT) in human bronchial epithelial cells and alveolar epithelial type II cells [20,38,39]. Another report indicates that aminophylline, which is a complex of the non-selective PDE inhibitor TEO and ethylenediamine, prevents airway EMT in Brown Norway rats after repeated allergen challenge [40]. Literature data also report a synergistic activity of a PDE4 and PDE5 inhibitors in reversing TGF-β-induced EMT in alveolar epithelial type II cells isolated from healthy donors [41]. The data presented in this study prove that pan-PDE inhibitors represent an interesting approach in reducing TGF-β-induced pro-mesenchymal changes in A549 cells. Our experiments revealed a very weak effect of TEO as a reference compound, on TGF-β-induced epithelial cell hallmarks. At the same time, 7,8-disubstituted TEO derivatives, compounds **1**, **2**, and **3**, significantly inhibited both a TGF-β-induced mesenchymal phenotype and migration of alveolar epithelial type II cells. The obtained results indicate that the modifications of the parent TEO structure, aimed at obtaining compounds with lower IC_50_ values in relation to individual PDE isoforms, translated to the stronger activity of representative 8-arylalkylamino- and 8-alkoxy-1,3-dimethyl-2,6-dioxo-1,2,3,6-tetrahydro-7*H*-purin-7-yl-*N*-(5-(*tert*-butyl)-2-hydroxyphenyl)butanamides against epithelial cells induced to acquire mesenchymal characteristics. As described here, beneficial **1**, **2**, and **3** activity may arise as a result of several overlapping aspects. First of all, our previously published data confirmed a prominent inhibitory activity of **1**, **2**, and **3** against PDE1, PDE3, PDE4, PDE5, PDE7, and PDE8 (Figure 2), the most important isoforms involved in the development of airway remodeling and lung fibrosis. This is important in the context of diverse PDE isoforms’ expression in bronchial epithelial cells [42,43]. Next, a study by Kolosionek et al. showed that in TGF-β-induced A549 cells, the expression of individual PDE types may also have changed. Some isoforms, e.g., PDE4A, PDE4D, and PDE8A, may be overexpressed and, conversely, the expression of some may be diminished [20]. Finally, considering the cAMP signaling compartmentalization during EMT [44], a different composition of individual isoforms within the cellular compartments cannot be ruled out. It has been postulated that molecules acting on multiple targets show superior efficacy against complex diseases that often require combination therapies, compared to compounds with high specificity for a single target [45]. They may not only potentiate efficacy additively or synergistically, but may also have a more predictable pharmacokinetic profile compared to several compounds administered in combination. Moreover, when using such compounds, the risk of drug–drug interactions associated with combination therapies is mitigated. Therefore, the search for new compounds that could simultaneously inhibit different PDE isoforms expressed in airways instead of using selective PDE inhibitors in combination seems rational and justified.

It is worth noting that all tested compounds within this study inhibited PDE3 or PDE4 at a similar level, but their activity against PDE7 or PDE8 was variable. Although all tested 8-substituted 1,3-dimethyl-2,6-dioxo-1,2,3,6-tetrahydro-7*H*-purin-7-yl-*N*- (5-(*tert*-butyl)-2-hydroxyphenyl)butanamides decreased TGF-β-induced mesenchymal features in A549 cells, derivatives **1** and **3** seem to be slightly more potent. This may be a consequence of their predominant impact on PDE8, an isoform lacking selective inhibitors, but which is considered to be a future drug target in inflammation and airway smooth muscle remodeling [46,47].

The fundamental role of the canonical TGF-β signaling through Smad proteins in the rising of a mesenchymal-like phenotype by epithelial cells has been well-understood and described [8,9]. It has also been demonstrated that inhibition of this signaling pathway may be crucial in reducing this phenomenon during airway remodeling and lung fibrosis. This kind of activity, by affecting Smad proteins, was proven, e.g., for apolipoprotein A1 [48], plant-derived hesperidin, paeoniflorin, and celasterol [49,50,51], as well as for two drugs approved in the treatment of idiopathic pulmonary fibrosis, nintedanib and pirfenidone [52,53]. With regard to compounds increasing the intracellular cAMP level and/or PDE inhibitors, there are several reports indicating an inhibitory effect of these compounds on the TGF-β-Smad signaling pathway. Fehrholz et al. showed that both the increased intracellular cAMP and the use of caffeine, a non-selective PDE inhibitor, as well as rolipram, a PDE4-selective one, inhibited TGF-β-induced Smad signaling in lung epithelial cells [54]. It was also shown that roflumilast *N*-oxide, an active metabolite of roflumilast (drug approved for COPD treatment) with potent, selective PDE4 inhibitory activity, significantly reduced TGF-β-Smad signaling in both human bronchial epithelial cells and alveolar epithelial type II cells [20,38]. Here, we displayed that representative 8-arylalkylamino- and 8-alkoxy-1,3-dimethyl-2,6-dioxo-1,2,3,6-tetrahydro-7*H*-purin-7-yl-*N*-(5-(*tert*-butyl)-2-hydroxyphenyl)butanamides significantly reduced the TGF-β-induced Smad-2 signaling in A549 cells by affecting both phosphorylation and the translocation of p-Smad-2 to the nucleus. To our knowledge, this is the first report demonstrating mesenchymal phenotype-limiting activity of pan-PDE inhibitors by decreasing Smad-2 signaling. At the same time, we added further evidence that targeting the TGF-β-Smad signaling pathway might represent the first choice in the design and development of new anti-fibrotic compounds.

The need for new and effective compounds that can be used in the therapy of chronic lung diseases still represents a major scientific challenge. On the one hand, thanks to the currently available pharmacotherapy, chronic airway inflammation or bronchospasm can be effectively controlled. Contrarily, prevention or inhibition of airway remodeling and lung fibrosis is a serious therapeutic problem. In addition, clinical data indicate that there is a group of asthma and COPD patients who exhibit corticosteroid insensitivity and do not respond to standard therapy [55,56]. Lately, it has been revealed that PDE4 inhibitors may be helpful in solving the problem of corticosteroid insensitivity. Roflumilast *N*-oxide has been shown to restore corticosteroid sensitivity in neutrophils, CD8 cells, and pulmonary endothelial cells [57,58,59]. These reports confirm that PDE inhibitors represent an interesting therapeutic alternative not only because of their promising anti-inflammatory and/or anti-fibrotic properties, but also because of their possible use in combination therapies. From this perspective, pan-PDE inhibitors appear to be of particular interest.

## 4. Materials and Methods

### 4.1. Compounds

The investigated compounds: 4-(8-(benzyl(methyl)amino)-1,3-dimethyl-2,6-dioxo-1,2,3,6-tetrahydro-7*H*-purin-7-yl)-*N*-(5-(*tert*-butyl)-2-hydroxyphenyl)butanamide (**1**), *N*-(5-(*tert*-butyl)-2-hydroxyphenyl)-4-(8-((2-methoxybenzyl)amino)-1,3-dimethyl-2,6-dioxo-1,2,3,6-tetrahydro-7*H*-purin-7-yl)butanamide (**2**), and 4-(8-butoxy-1,3-dimethyl- 2,6-dioxo-1,2,3,6-tetrahydro-7*H*-purin-7-yl)-*N*-(5-(*tert*-butyl)-2-hydroxyphenyl)butanamide (**3**), were synthesized following a multistep procedure (Supplementary Schemes S1 and S2) according to previously described methods [21,23,60].

1,3-Dimethyl-3,7-dihydro-1*H*-purine-2,6-dione (TEO) was used as a control in the study. All tested compounds were dissolved in dimethyl sulfoxide (DMSO; Sigma Aldrich, St. Louis, MO, USA), and the final DMSO concentration in culture medium did not exceed 0.5%. At this concentration, the solvent had no harmful effect on the cells. The IC_50_ of **1**–**3** were published previously [23,25], except PDE8A IC_50_ for TEO, which was determined as described in [23], using hrPDE8A (SignalChem, Richmond, Canada) and the PDE-Glo™ catalytic activity assay with a luminescence detection (Promega Corporation, Madison, WI, USA).

### 4.2. Cell Culture

The A549 cells (ATCC^®^ CCL-185™, Manassas, VA, USA) were cultured in Ham’s F-12K (Kaighn’s) Medium (Gibco, Thermo Fisher Scientific, Waltham, MA, USA) supplemented with 10% (*v*/*v*) fetal bovine serum (FBS; Gibco, Thermo Fisher Scientific, Waltham, MA, USA) and an antibiotics mixture (penicillin, streptomycin, amphotericin B; Gibco, Thermo Fisher Scientific, Waltham, MA, USA) in standard culture conditions (5% CO_2_, 37 °C, 95% humidity). The mesenchymal phenotype in alveolar epithelial type II cells was induced in serum-depleted medium (1%; *v*/*v*) by the addition of TGF-β (5 ng/mL; BD Biosciences, San Jose, CA, USA). The investigated compounds were administrated 1 h before TGF-β treatment. Depending on the analyzed endpoint, cells were cultured in the presence of compounds and/or TGF-β for 1, 24, or 48 h.

### 4.3. Cell Morphology, Viability, and Proliferation

Cell morphology was examined using an inverted bright-field microscope, Leica DMi1 (20x objective, Leica Microsystems GmbH, Wetzlar, Germany). Changes in cell morphology under the influence of tested compounds were examined based on the collected microscopic images. Cell elongation index was calculated using the following formula: cell elongation index = (major axis − minor axis)/(major axis + minor axis). A549 cells’ viability was performed in the MTT (3-(4,5-dimethylthiazol-2-yl)-2,5-diphenyltetrazolium bromide) assay, after 48 h of incubation with the investigated compounds (0.5–100 μM). MTT reagent was added at a final concentration of 0.5 μg/mL. After dissolving formazan crystal in DMSO, the absorbance at 570 nm was measured with a multi-functional microplate reader (SpectraMax^®^ iD3, Molecular Devices, San Jose, CA, USA). A549 cell proliferation rate was determined using the crystal violet staining. Tested compounds were administrated at growing concentrations (1–50 μM) 1 h prior to FBS (10%, *v*/*v*) 48 h incubation. Then, cells were fixed in 3.7% formaldehyde solution, incubated with 0.5% crystal violet solution (Sigma Aldrich, St. Louis, MO, USA), and after de-staining in citric acid/sodium citrate mixture, the supernatant absorbance was measured at 570 nm using a microplate reader. Both viability and proliferation assays were run four times in duplicates.

### 4.4. Migration Assays

A549 migration was evaluated using Transwell and wound healing assays. In the first method, 6.5 mm Transwell culture plates with an 8.0 µm pore polycarbonate membrane (Corning Incorporated, NY, USA) were used. After serum starvation, cells were seeded in the Transwell upper chamber in serum-depleted medium with or without study compounds (10 μM). The bottom Transwell part was filled with medium supplemented with TGF-β. After 24 h of incubation, cells were fixed with 4% formaldehyde solution (Sigma Aldrich, St. Louis, MO, USA) and stained with 0.5% crystal violet solution (Sigma Aldrich, St. Louis, MO, USA). Cells from the upper side of the polycarbonate membrane were removed using a cotton swab. The number of migrated cells was counted under an inverted microscope (Nikon Eclipse TS 100, 10× objective) using 10 randomly selected fields of view. Experiments were run three times, in a blindfolded manner. For the wound healing assay, cells were seeded in 6-well plates and cultured until confluency. Then, a straight scratch was made with a 20–200 μL sterile pipette tip in the cell monolayer, and serum-depleted culture medium supplemented with or without study compounds (10 μM) was added 1 h before TGF-β treatment. After 0 and 48 h of A549 incubation with TGF-β, microphotographs were taken using an inverted microscope, Leica DMi1 (Leica Microsystems GmbH, Wetzlar, Germany). Experiments were run three times, in a blindfolded manner. Wound healing rate was calculated based on the analysis of 10 randomly selected fields of view by using Fiji ImageJ software, version 1.53e.

### 4.5. Immunofluorescence

To visualize vimentin and p-Smad-2 in A549 cells, an immunofluorescence staining was used. Additionally, visualization of the F-actin cytoskeleton was performed. Cells were seeded on collagen-coated glass coverslips. Vimentin and F-actin were detected after 48 h, and p-Smad-2 was detected after 1 h of incubation with TGF-β. Briefly, cells were fixed in 4% formaldehyde solution, permeabilized in 0.2% Triton X-100 solution, and incubated in 3% bovine albumin serum (BSA; Sigma Aldrich, St. Louis, MO, USA) blocking solution. Then, incubation with rabbit monoclonal, anti-vimentin antibody (#MA5-14564, Thermo Fisher Scientific, Waltham, MA, USA) and rabbit polyclonal, anti-phospho-Smad-2 (Ser465, Ser467) antibody (#44-244G, Thermo Fisher Scientific, Waltham, MA, USA), followed by goat anti-rabbit Alexa Fluor 546 conjugated secondary antibody (#A-11010, Invitrogen, Carlsbad, CA, USA), was performed. F-actin cytoskeleton was detected by incubation with TRITC-labeled phalloidin solution (Sigma Aldrich, St. Louis, MO, USA). Nuclei were counterstained with Hoechst 33342 dye (Thermo Fisher Scientific, Waltham, MA, USA). Finally, slides were mounted in ProLong™ Glass Antifade Mountant (Invitrogen, Carlsbad, CA, USA) and analyzed using a Leica DMiL LED Fluo microscope (40× objective) equipped with LAS-X Software (version 3.0.4.16529, Leica Microsystems GmbH, Wetzlar, Germany). Experiments were run three times, at the same fluorescent time exposure and in a blindfolded manner. Quantitative analysis of the percentage of cells representing nuclear p-Smad-2 localization was performed based on the analysis of 10 independent fields of view in each individual experiment.

### 4.6. ELISA

The in-cell ELISA method was used to measure the relative content of vimentin, α-SMA, and collagen I in A549 cells. Cells were fixed in ice-cold methanol, permeabilized, and blocked in 1% bovine serum albumin (BSA) with 0.1% Tween^®^ 20 and then immunostained with primary rabbit monoclonal anti-vimentin, mouse monoclonal anti-α-SMA, and mouse monoclonal anti-collagen type I antibodies (#MA5-14564, #A2547, and #SAB4200678, respectively, Thermo Fisher Scientific, Waltham, MA, USA, and Sigma Aldrich, St. Louis, MO, USA), followed by secondary anti-rabbit or anti-mouse, peroxidase-conjugated antibody (#32460, Thermo Fisher Scientific, Waltham, MA, USA and #A9044, Sigma Aldrich, St. Louis, MO, USA, respectively). Finally, a colorimetric reaction was induced by the addition of tetramethylbenzidine (Sigma-Aldrich, St. Louis, MO, USA). After sufficient color development, 1N HCl was added and the absorbance was measured at 450 nm. The experiments were run four times in duplicates.

### 4.7. Western Blot Analysis

Western Blot analysis was performed to analyze p-Smad-2 and p-CREB levels. Total protein from the cell culture was extracted using RIPA buffer (RIPA Lysis and Extraction Buffer, Thermo Fisher Scientific, Waltham, MA, USA) supplemented with protease inhibitor cocktail and phosphatase inhibitor cocktail (both from Sigma Aldrich, St. Louis, MO, USA). Next, equal amounts of protein (30 µg) were loaded in reducing sample buffer (4% SDS, 20% glycerol, 10% 2-mercaptoethanol, 0.004% bromphenol blue, and 0.125 M Tris-HCl, pH 6.8) onto a 10% polyacrylamide gel for electrophoresis. Separated protein was transferred onto the polyvinylidene difluoride (PVDF) membrane (Thermo Fisher Scientific, Waltham, MA, USA), washed with Tris Buffered Saline with Tween 20 (TBST) buffer (150 mM NaCl, 50 mM Tris, 0.05% Tween 20), and blocked with 5% skim milk in TBST. To detect a specific protein of interest, membranes were incubated overnight with primary antibodies: rabbit, polyclonal anti-SMAD2 antibody, dilution 1:250, #51-1300, rabbit, polyclonal anti-phospho-SMAD2 (Ser465, Ser467) antibody, dilution 1:500, #44-244G (both from Thermo Fisher Scientific, Waltham, MA, USA), rabbit, polyclonal anti-phospho-CREB1 (Ser133) antibody, dilution 1:500, #orb213775 (Biorbyt LLC, San Francisco, CA, USA), and mouse monoclonal anti-GAPDH antibody, dilution 1:5000, #G8795 (Sigma Aldrich, St. Louis, MO, USA), followed by secondary rabbit anti-mouse IgG (whole molecule) peroxidase antibody (#A9044, dilution 1:3000, Sigma Aldrich, St. Louis, MO, USA) and goat anti-rabbit IgG (H + L) peroxidase-conjugated antibody (#32460, dilution 1:3000, Thermo Fisher Scientific, Waltham, MA, USA). Proteins were detected using a chemiluminescence method (Clarity Max™ Western ECL Substrate—Peroxide Solution, BioRad, Hercules, CA, USA) with a C-DiGit^®^ Blot Scanner (LI-COR Biosciences, Lincoln, NE, USA). ROD was measured according to the NIH guidelines with Fiji ImageJ Software, version 1.53e. The experiments were run four times.

### 4.8. Gelatin Zymography

Gelatin zymography was performed to detect MMP9 and MMP2 activity in cell culture supernatants. Collected supernatants were diluted in non-reducing sample buffer (4% SDS, 20% glycerol, 0.01% bromophenol blue, 125 mM Tris-HCl, pH 6.8) and then subjected to electrophoresis in 10% SDS-polyacrylamide containing gelatin from porcine skin (1 mg/mL, Sigma-Aldrich, St. Louis, MO, USA). After electrophoresis, gels were washed to remove SDS and incubated overnight in digestion buffer containing cofactors necessary for the gelatinase reaction (1% Triton X-100, 50 mM Tris-HCl, pH 7.5, 5 mM CaCl_2_, 1 µM ZnCl_2_). Next, gels were fixed and stained with Coomasie brilliant blue (Sigma-Aldrich, St. Louis, MO, USA). To visualize the areas of gelatin digestion, the gels were rinsed in de-staining solution. The obtained clear bands against a dark blue background were then compared with protein standard and analyzed using Fiji ImageJ software, version 1.53e. The experiments were run five times.

### 4.9. RNA Extraction and Reverse Transcription-Quantitative PCR

Reverse transcription-quantitative polymerase chain reaction (RT-qPCR) was performed to analyze the expression level of selected genes. The total RNA was extracted using the Total RNA Mini Kit (A&A Biotechnology, Gdynia, Poland) according to the manufacturer’s protocol. Next, equal amounts of total RNA (about 1 µg) were reverse transcribed using the iScript™ cDNA Synthesis Kit (BioRad, Hercules, CA, USA). qPCR was performed using the CFX96 Touch Real-Time PCR Detection System (BioRad, Hercules, CA, USA) and TaqMan^®^ Fast Advanced Master Mix (Applied Biosystems, Foster City, CA, USA), with specific probes for: *CDH1* (Hs01023895_m1), *CDH2* (Hs00983056_m1), *SNAI1* (Hs00195591_m1), *VIM* (Hs00958111_m1), *FN1* (Hs01549976_m1), *TGFB1* (Hs00998133_m1), and *MMP9* (Hs00957562_m1). qPCR cycling (40x) was performed with denaturation at 95 °C for 3 s and annealing/extension at 60 °C for 30 s. The relative abundance of specific mRNA transcripts was estimated based on the cycle threshold (Ct) values and recalculated against the endogenous reference *18S* (Hs99999901_s1) ribosomal RNA using the ∆∆Ct method. The experiments were run three times in duplicates.

### 4.10. Data Analysis

Statistical analysis was performed with GraphPad Prism (GraphPad Software, Inc., San Diego, CA, USA, version 5.01). Values presented in the graphs correspond to the mean ± standard deviation (SD), except where stated otherwise. The normality of distribution was estimated using the Shapiro–Wilk test. The statistical significance of differences between means of normally distributed data was determined by ANOVA with Dunnett’s post-hoc test. When it was not possible to determine the Gaussian distribution of the data, the statistical significance of differences was determined using the Kruskal–Wallis test with Dunn’s post-hoc test. Differences were considered to be statistically significant at *p* < 0.05.

## 5. Conclusions

Based on both the previous results and those presented within this work, we argued that pan-PDE inhibitors represent promising anti-inflammatory and anti-fibrotic agents. Here, we described a new and interesting activity of 8-arylalkylamino- and 8-alkoxy-1,3-dimethyl-2,6-dioxo-1,2,3,6-tetrahydro-7*H*-purin-7-yl-*N*-(5-(*tert*-butyl)-2-hydroxyphenyl)butanamides, by their influence on the expression of several mesenchymal markers and augmented alveolar epithelial type II cells’ migration, thus adding another feature to their anti-fibrotic pharmacological profile. Considering the multifunctional activity of pan-PDE inhibitors in inflammation and fibrosis mitigation, it seems that designing new compounds targeting lung diseases not only in the group of 1,3-dimethyl-3,7-dihydro-1*H*-purine-2,6-dione derivatives but also within other chemical groups may bring many therapeutic benefits in the future.

## Data Availability

Data is contained within article and Supplementary Materials.

## Supplementary Materials

The following supporting information can be downloaded at: https://www.mdpi.com/article/10.3390/ph15040423/s1, Figure S1: Original gels of the data reported in Figure 5F; Figure S2: Original Western blot membranes of the data reported in Figure 6A,B; Scheme S1: Synthesis of compound **1** and **2**, [23]; Scheme S2: Synthesis of compound **3**, [21,60].
