# Supplementary material for: Pan-Phosphodiesterase Inhibitors Attenuate TGF-β-Induced Pro-Fibrotic Phenotype in Alveolar Epithelial Type II Cells by Downregulating Smad-2 Phosphorylation
Source: Pharmaceuticals (Basel). 2022 Mar 30;15(4):423. doi: 10.3390/ph15040423 (PMC9024446; doi:10.3390/ph15040423)
Supplement: Supplementary file 1 [file pharmaceuticals-15-00423-s001.zip › pharmaceuticals-1627987-SM.pdf]

Pan-Phosphodiesterase Inhibitors Attenuate TGF-β-Induced Pro-Fibrotic Phenotype in Alveolar Epithelial Type II Cells by Downregulating Smad-2 Phosphorylation

Katarzyna Wójcik-Pszczoła [1,*], Grażyna Chłoń-Rzepa [2], Agnieszka Jankowska [2], Bruno Ferreira [1,†], Paulina Koczurkiewicz-Adamczyk [1], Elżbieta Pękala [1], Elżbieta Wyska [3], Krzysztof Pociecha [3] and Reinoud Gosens [4]

[1] Department of Pharmaceutical Biochemistry, Faculty of Pharmacy, Jagiellonian University Medical College, Medyczna 9, 30-688 Kraków, Poland; bruno.fer.9719@gmail.com (B.F.); paulina.koczurkiewicz@uj.edu.pl (P.K.-A.); elzbieta.pekala@uj.edu.pl (E.P.)

[2] Department of Medicinal Chemistry, Faculty of Pharmacy, Jagiellonian University Medical College, Medyczna 9, 30-688 Kraków, Poland; mfchlon@cyf-kr.edu.pl (G.C.-R.); agnieszkajankowska@poczta.onet.pl (A.J.)

[3] Department of Pharmacokinetics and Physical Pharmacy, Faculty of Pharmacy, Jagiellonian University Medical College, Medyczna 9, 30-688 Kraków, Poland; mfwyska@cyf-kr.edu.pl (E.W.); k.pociecha@uj.edu.pl (K.P.)

[4] Department of Molecular Pharmacology, University of Groningen, Antonius Deusinglaan 1, 9713 AV Groningen, The Netherlands; r.gosens@rug.nl

* Correspondence: katarzynaanna.wojcik@uj.edu.pl; Tel.: +48-12-620-55-77

† Bruno Ferreira was a student of the Erasmus+ Program, from Faculty of Pharmacy, University of Porto, 4099-002 Porto, Portugal.

Supplementary data

Synthesis of tested compounds 1-3.

The previously described [23] compounds **1** (**32,** [23]) and **2** (**35,** [23]) were synthesized according to a multistep procedure presented in Scheme 1. Briefly, to obtain the compound **1** (**32,** [23]), in the first step of the synthesis, 8-bromo-1,3-dimethyl-3,7- dihydro-1*H*-purine-2,6-dione (8-BrTEO) was treated with *N*-methyl-1-phenylmethanamine in refluxing 2-methoxyethanol to obtain 8-(benzyl(methyl)amino)-1,3-dimethyl- 3,7-dihydro-1*H*-purine-2,6-dione (**1,** [23]). Next, this intermediate was alkylated at position 7 using ethyl 4-bromobutanoate in the presence of K_2CO_3 and catalytic amount of *N*-benzyl-*N,N*-diethylethanaminium chloride (TEBA) in refluxing acetone. The obtained ethyl 4-(8-(benzyl(methyl)amino)-1,3-dimethyl-2,6-dioxo-1,2,3,6-tetrahydro-7*H*-purin- 7-yl)butanoate (**9,** [23]) was hydrolyzed using KOH in water-acetone mixture and acidified with concentrated HCl to yield the corresponding 4-(8-(benzyl(methyl)amino)-1,3-dimethyl-2,6-dioxo-1,2,3,6-tetrahydro-7*H*-purin-7-yl)butanoic acid (**17,** [23]). In the final step, the synthesized acid was condensed with 2-amino-4-*tert*-butylphenol in *N,N*-dimethylformamide (DMF) using di(1*H*-imidazol-1-yl)methanone (CDI) as an activating agent to yield 4-(8-(benzyl(methyl)amino)-1,3-dimethyl-2,6-dioxo-1,2,3,6-tetrahydro-7*H*-purin-7-yl)-*N*-(5-(*tert*butyl)-2-hydroxyphenyl)butanamide (**32,** [23]). Compound **2** (**35,** [23]) was obtained analogously using 1-(2-methoxyphenyl)-methanamine in the first step of the synthesis. The appropriate intermediate products are as follow: 8-((2-methoxybenzyl)amino)-1,3-dimethyl-3,7-dihydro-1*H*-purine-2,6-dione **4,** [23], 4-(8-((2-methoxybenzyl) amino)-1,3-dimethyl-2,6-dioxo-1,2,3,6-tetrahydro-7*H*-purin7-yl) -2,6-yl)butanoate **12,** [23] and 4-(8-((2-methoxybenzyl) amino)-1,3-dimethyl-2,6-dioxo- 1,2,3,6-tetrahydro-7*H*-purin7-yl)-2,6-yl)butanoic acid **20** **[23]**. In the final step the acid **20,** [23] was condensed with 2-amino-4-*tert*-butylphenol to yield compound **2** (**35,** [23]).

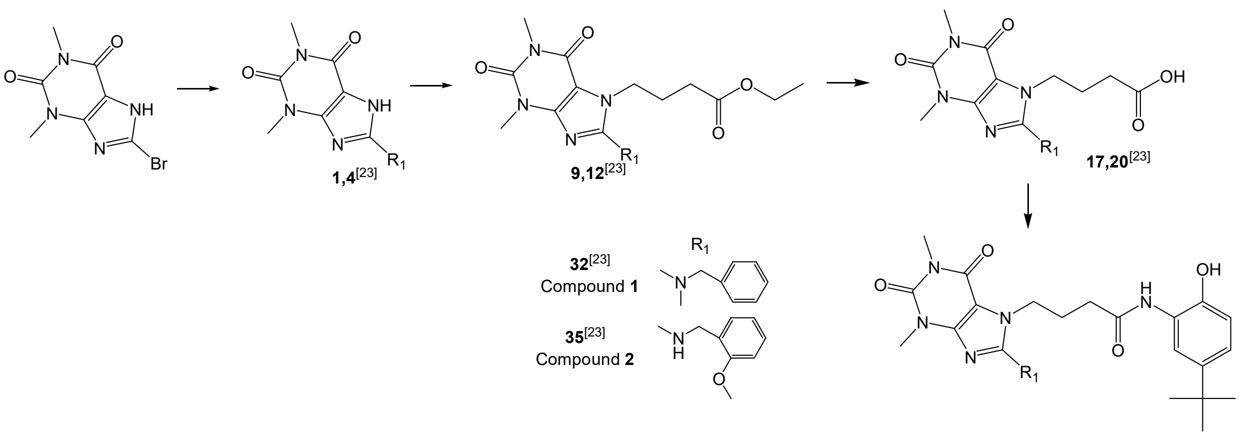

Scheme S1. Synthesis of compound 1 and 2. Full characteristics of intermediates and final products was described in [23]. The numbers assigned to the compounds shown in the scheme correspond to the respective numbers and chemical names presented in the original manuscript [23].

Compound **3** (**36,** [21]) was synthesized according to a multistep procedure described in [21] and presented in Scheme 2. The 8-BrTEO was first alkylated at position 7 using ethyl 4-bromobutanoate in the presence of K_2CO_3 and catalytic amount of TEBA in refluxing acetone yielding ethyl 4-(8-bromo-1,3-dimethyl-2,6-dioxo-1,2,3,6-tetrahydro- 7*H*-purin-7-yl)butanoate (**2,** [60]), which was then treated with sodium butanolate in butanol to obtain butyl 4-(8-butoxy-1,3-dimethyl-2,6-dioxo-1,2,3,6-tetrahydro- 7*H*-purin-7-yl)butanoate (**8,** [21]). Next, the resulting ester was subjected to alkaline hydrolysis. After acidification the obtained 4-(8-butoxy-1,3-dimethyl-2,6-dioxo-1,2,3,6- tetrahydro-7*H*-purin-7-yl) butanoic acid (**14,** [21]) was finally condensed with 2-amino-4-*tert*-butylphenol in similar conditions as describe above to give **3** (**36,** [21]).

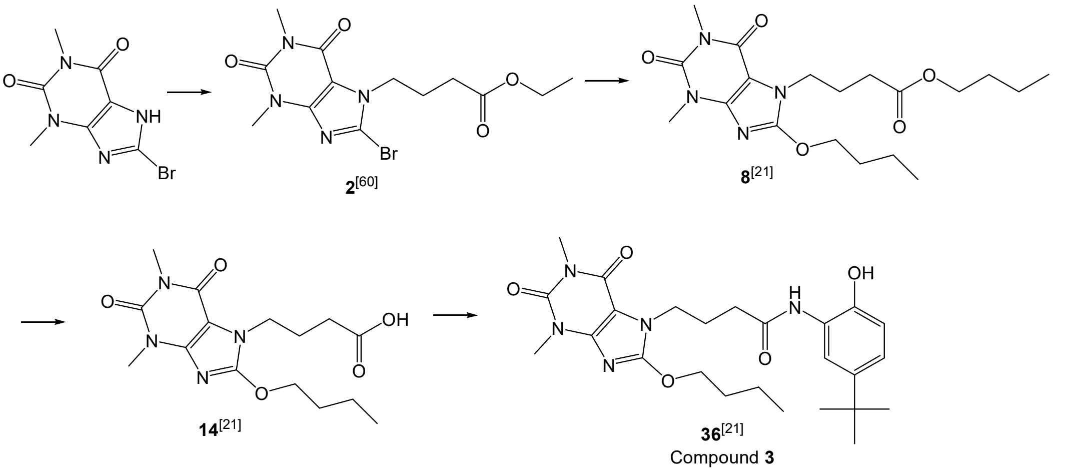

Scheme S2. Synthesis of compound 3. Full characteristics of intermediates and final products was described in [21, 60]. The numbers assigned to the compounds shown in the scheme correspond to the respective numbers and chemical names presented in the original manuscript [21, 60].

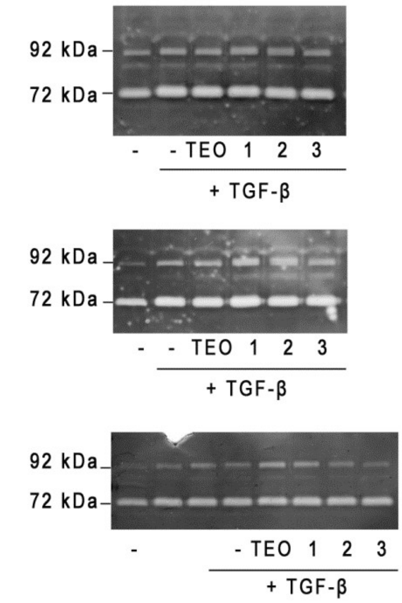

Figure S1. Original gels of the data reported in Fig. 5F.

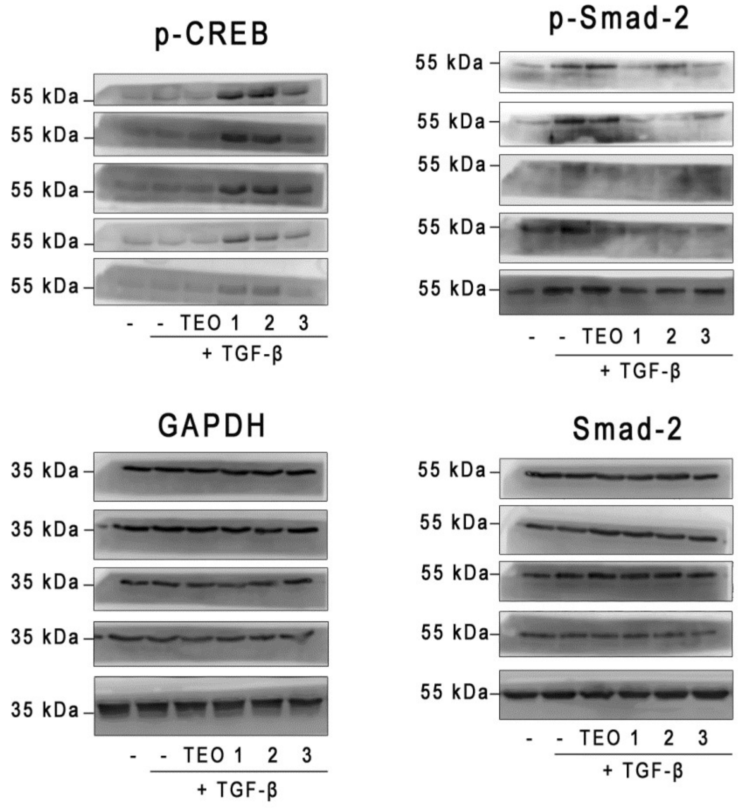

Figure S2. Original Western blot membranes of the data reported in Fig. 6A-B.

References

21. Chłoń-Rzepa, G.; Ślusarczyk, M.; Jankowska, A.; Gawalska, A.; Bucki, A.; Kołaczkowski, M.; Świerczek, A.; Pociecha, K.; Wyska, E.; Zygmunt, M.; et al. Novel amide derivatives of 1,3-dimethyl-2,6-dioxopurin-7-yl-alkylcarboxylic acids as multifunctional TRPA1 antagonists and PDE4/7 inhibitors: A new approach for the treatment of pain. Eur. J. Med. Chem. **2018**, 158, 517–533.
23. Wójcik-Pszczoła, K.; Jankowska, A.; Ślusarczyk, M.; Jakieła, B.; Plutecka, H.; Pociecha, K.; Świerczek, A.; Popiół, J.; Koczurkiewicz-Adamczyk, P.; Wyska, E.; et al. Synthesis and in vitro evaluation of anti-inflammatory, antioxidant, and anti-fibrotic effects of new 8-aminopurine-2,6-dione-based phosphodiesterase inhibitors as promising anti-asthmatic agents. Bioorg. Chem. **2021**, 117, 105409.
60. Pawłowski, M.; Chłoń-Rzepa, G.; Obniska, J.; Zejc, A.; Charakchieva-Minol, S.; Mokrosz; M.J. Synthesis 5HT1A and 5-HT2A receptor affinity of new 1-phenylpiperazinylpropyl derivatives of purine-2,6- and pyrrolidine-2,5-diones. Il Farmaco. 2000, 55, 461-468.
